# Relationship between epicardial adipose tissue thickness and sedentary time, physical activity level, and physical performance in patients with hypertension

**DOI:** 10.1038/s41371-025-01002-y

**Published:** 2025-03-11

**Authors:** Emine Tunc Suygun, Naciye Vardar Yagli, Hakan Suygun

**Affiliations:** 1 Vocational School of Health Services, Department of Therapy and Rehabilitation, Karamanoğlu Mehmetbey University, Karaman, Turkey; 2 Faculty of Physical Therapy and Rehabilitation, Hacettepe University, 06100 Samanpazari, Ankara, Turkey; 3 Faculty of Medicine, Department of Cardiology, Karamanoğlu Mehmetbey University, Karaman Training and Research Hospital, Karaman, Turkey

**Keywords:** Risk factors, Hypertension

## Abstract

Epicardial adipose tissue is associated with the development of cardiovascular disease and its increase is positively correlated with blood pressure elevation in hypertensive individuals. In the literature, being physically active has been shown to be effective in the treatment of hypertension and reduction of epicardial adipose tissue thickness. The aim of this study was to evaluate the relationship between epicardial adipose tissue thickness and sedentary time, physical activity level and physical performance in patients with hypertension. The physical and demographic characteristics of the 40 patients with hypertension were collected with sociodemographic form. Waist/hip circumference was measured with tape measure and epicardial adipose tissue thickness with echocardiography device. Physical activity levels and sedentary time were recorded via IPAQ-7. Physical performance was determined using the 30-s sit-and-stand test. There was a moderate negative correlation between epicardial adipose tissue thickness and IPAQ-7 score (rho: −0.568 p < 0.001) and a high negative correlation between epicardial adipose tissue thickness and sit-and-stand test score (rho: −0.794 p < 0.001). There was no relationship between epicardial adipose tissue thickness and daily sitting time, BMI, or hip circumference. There was moderate positive correlation between epicardial adipose tissue thickness and age (rho: 0.504 p: 0.001) and low positive correlation between waist circumference (rho: 0.322 p < 0.05).This study demonstrated that the epicardial adipose tissue thickness was negatively associated with activity and performance in hypertensive patients, but not with daily sitting time. These results underscore the importance of physical activity in the management and prevention of chronic diseases.

## Introduction

The development of cardiovascular disease is associated with obesity and visceral adipose tissue accumulation as well as increased cardiac adipose tissue [1–3]. Epicardial adipose tissue (EAT), a cardiac visceral fat depot, has been shown to contribute to the development and progression of atrial fibrillation and heart failure with preserved ejection fraction [4]. In addition, it has been reported that EAT may be a marker auxiliary to classical risk factors for the presence and severity of coronary artery disease [5]. EAT thickness is also associated with metabolic syndrome and hypertension, high levels of low-density lipoprotein cholesterol, and insulin resistance [6–9].

The EAT is located between the myocardium and the pericardium and is therefore in direct contact with the main coronary arteries and their branches [10]. Under normal conditions, EAT is thought to be able to metabolize large amounts of fatty acids. Thus, it is suggested to play a cardioprotective role by preventing the formation of atherosclerotic plaques [1, 11]. Increased EAT has been shown to affect the cardiovascular system by increasing the release of inflammatory mediators that cause atherosclerosis and vascular endothelial dysfunction [2, 12, 13]. Studies have shown that the EAT of individuals diagnosed with hypertension is thicker than that of normotensive individuals and EAT thickness is positively correlated with blood pressure levels [9, 14].

Many studies have shown that regular physical activity, which is considered an effective non-pharmacological treatment method in the management of hypertension, is associated with lower blood pressure and lower incidence of hypertension [15, 16]. Our search of the literature revealed no study evaluating the relationship between physical activity and physical performance levels in hypertensive individuals and EAT thickness. The aim of the present study was to examine the relationship between EAT thickness and sedentary time, physical activity level, and physical performance in hypertensive patients.

## Methods

The study included 40 patients with a diagnosis of hypertension who presented to the cardiology outpatient clinics of Karaman Training and Research Hospital between December 6, 2021 and February 28,2022. Karamanoğlu Mehmetbey University Faculty of Medicine (Non-Interventional) Approval was obtained from the Ethics Committee (Ethics Committee Decision No 07- 2021/05 and ClinicalTrials.gov Identifier: NCT05890989). Before the study, the purpose and content of the study were explained to participants. Written informed consent was obtained from the participants stating that they participated in the study voluntarily.

### Participants

Volunteers who had been diagnosed with hypertension for at least 6 months, could speak Turkish, were literate, and aged 18–65 years were included in the study. Individuals with severe valvular disease, heart failure, cardiomyopathy, diabetes mellitus, chronic kidney disease, thyroid diseases, hyperlipidemia, active infectious or inflammatory diseases, or serious psychiatric diseases such as panic disorder or major depression, smoking were excluded. As a result of the power analysis, the sample size required to find a significant relationship of 50.9%, which was obtained as a result of the pilot study, between EAT thickness and physical activity level with 5% error level and 95% power was calculated as 37 individuals.

### Evaluation

The physical and demographic characteristics of the participants (age, sex, height, body weight, body mass index (BMI), educational status, employment status, duration of hypertension disease, medications, and comorbidity) were recorded. Waist/hip circumference measurements were recorded by the same investigator using a tape measure. Office blood pressure (BP) was measured after 5 min of rest in a quiet environment using a oscillometric device with the upper arm cuff with the patient in a sitting position. Patients were advised to refrain from smoking, physical exercise, and consumption of coffee and tea 30 min before BP measurements.

All patients underwent transthoracic echocardiography conducted by a single cardiologist using a Philips HD11XE (Philips Medical Systems) device in the left lateral decubitus position by the standard technique. Epicardial fat thickness was measured in the parasternal long axis imaging window, using the aortic annulus as an anatomical indicator for vertical measurement, and the widest part of the region from the right ventricular free wall to the pericardium at the end of the systole [17].

The physical performance of the participants was evaluated with the 30-s sit-and-stand test. While seated in a chair with a seat height of 43.2 cm and a supported back, the patient was asked to stand up and sit down as quickly as possible while crossing his/her hands over his/her chest. The 30-s duration of the test was monitored with a stopwatch and the number of times the participant stood up and sat down was noted. Two trials were performed before the test [18].

Physical activity level was evaluated with the Turkish version of the International Physical Activity Questionnaire-7 (IPAQ-7), whose reliability and validity were confirmed by Saglam et al. [19]. The IPAQ-7 is a seven-item questionnaire that assesses severe physical activity (duration: min and frequency: day), moderate physical activity (duration: min and frequency: day), and walking time of at least 10 min (frequency: day) over the previous seven days. The metabolic equivalent of task (MET) corresponding to the basal metabolic rate is converted from severe and moderate activity and walking periods, and the overall physical activity score (MET- min/week) is determined.

### Statistical analysis

The statistical analyses were performed with IBM SPSS Statistics V23. The data were analyzed using normality tests (Shapiro–Wilk test for n < 50 and Kolmogorov–Smirnov test for n ≥ 50) and graphs such as histograms and qq plots. The relationships between variables were analyzed by Pearson’s correlation coefficient for normally distributed variables and Spearman’s correlation coefficient and multiple linear regression analysis for non-normally distributed variables. Comparisons between two independent groups were analyzed with the significance test of the difference between two means (independent t-test) when the parametric test assumptions were met, and with the Mann–Whitney U test when they were not. Comparisons between more than two independent groups were analyzed by one-way ANOVA when parametric test assumptions were met and by Kruskal–Wallis test when not met. Continuous variables in the physical and demographic data are expressed as mean (X) ± standard deviation (SD) and median (minimum- maximum) depending on the distribution. Categorical variables, such as sex, are expressed as numbers (%).

## Results

The physical and demographic findings of the patients included in the study are presented in Table 1. The median hypertension duration was 42 (min-max = 12–240) months.

**Table 1:** Physical and demographic findings and evaluation results of the participants.

Age (year, mean ± SD)	50 ± 9
BMI (kg/m^2^, mean ± SD)	30.70 ± 4.58
Anthropometric Measurements	
Waist circumference (cm, mean ± SD)	103 ± 10
Hip Circumference (cm, median [min-max])	110 (96–140)
Blood pressure measurements (Office)	
Systolic BP (mmHg, mean ± SD)	130,80 ± 6,25
Diastolic BP (mmHg, mean ± SD)	84,92 ± 3,30
Heart Rate (bpm)	77,87 ± 6,84
Sex (n, %)	
Female	30 (75%)
Male	10 (25%)
Working status (n, %)	
Working	14 (35%)
Not working	26 (65%)
Education status (n, %)	
Primary school	25 (62.5%)
Secondary school	2 (5%)
High school	8 (20%)
University	5 (12.5%)
Medication (n, %)	
ACE inhibitors + Diüretics	18 (45%)
Calcium channel blocker	5 (12,5%)
ACE inhibitors + Calcium channel blocker	8 (20%)
ARB+ Calcium channel blocker	9 (22,5)
Beta-Blocker, n (%)	2 (5%)

*BP* blood pressure, *ACE* angiotensin-converting enzyme, *ARB* angiotensin recep- tor blocker, *Min* minimum, *max* maximum, *SD* standard deviation.

The IPAQ-7 and sit-and-stand test scores, EAT thickness, and hours spent sitting during the day of the patients included in the study are presented in Table 2.

**Table 2:** Participants’ evaluation results.

IPAQ-7 (MET, median [min-max])	1464 (330–10,344)
EAT (mm, median [min-max])	6.35 (4.50–10.60)
Sit-and-stand test score (mean ± SD)	15.20 ± 2.68
Hours spent sitting during the day (hours, median [min-max])	8 (4–10)

*IPAQ* international physical activity questionnaire-7, *EAT* epicardial adipose tissue, *min* minimum, *max* maximum, *SD* standard deviation.

There was a moderate negative correlation (rho: −0.568 p < 0.001) between EAT thickness and IPAQ-7 score and a high negative correlation (rho: −0.794 p < 0.001) between EAT thickness and sit-and-stand test score. There was no statistically significant relationship between EAT thickness and daily sitting time, BMI, or hip circumference. There was a moderate positive correlation between EAT thickness and age (rho: 0.504 p: 0.001) and a low positive correlation between EAT thickness and waist circumference (rho: 0.322 p < 0.05) (Table 3).

**Table 3:** The relationship between EAT and IPAQ-7, sit-and-stand test score, daily sitting time, age, BMI, and anthropometric measurements.

Variable	IPAQ-7	Sit-and-Stand	Daily Sitting Time	Age	BMI	Waist Circumference	Hip Circumference
EAT thickness	rho: −0.568	rho: −0.794	rho: 0.077	rho: 0.504	rho: 0.240	rho: 0.322	rho: 0.122
	p: 0.000	p: 0.000	p: 0.636	p: 0.001	p: 0.136	p: 0.043	p: 0.454

*IPAQ* international physical activity questionnaire-7, *BMI* body mass index.

p < 0.05.

As a result of the multiple regression analysis performed with the model consisting of the variables determined to have statistically significant relationships in the analyses and the EAT thickness variable, the IPAQ-7 score, sit-and-stand test score, age, and waist circumference were determined as independent-explanatory variables. However, the presence of high correlations between the independent variables (for example, there is a 75.9% correlation between IPAQ-7 score and sit-and-stand score) affected the significance of all variables in the model. Therefore, only significant variables were left in the model by using stepwise variable selection. The independent variables that provided model fit are shown in Table 4. The regression model obtained as a result of the analysis was universally significant (F = 28.478, p < 0.001). Sit-and-stand score and waist circumference together can explain 58.5% of the change in EAT thickness. As the sit-and-stand score increases by 1 unit, EAT thickness decreases by 0.439 mm on average. As waist circumference increases by 1 unit, EAT thickness increases by 0.036 mm. According to the beta coefficients, sit-and-stand score was more influential on EAT than waist circumference was.

**Table 4:** Regression analysis results for the effect of sit-and-stand score and waist circumference on EAT thickness.

	Unstandardized Coefficients		Standardized Coefficients	t	p	95.0% Confidence Interval for B		
Variables	B	Std. Error	Beta			Lower Bound	Upper Bound	
Constant	9.755	2.030		4.804	0.000	5.641	13.869	R^2^: 0.585 F: 28.478 p < 0.001
Sit-and-stand score	−0.439	0.063	−0.721	−6.953	**0.000**	−0.566	−0.311	
Waist circumference	0.036	0.016	0.227	2.184	**0.035**	0.003	0.069	

R^2^: Explanatory coefficient.

## Discussion

To the best of our knowledge, this is the first study to evaluate the relationship between EAT thickness and sedentary time, physical activity level, and physical performance in patients with hypertension. Our study showed that EAT thickness was negatively associated with physical activity and physical performance in these patients. No relationship was found between EAT thickness and daily sitting time.

The negative correlation in our study between physical activity level and EAT thickness in hypertensive individuals may be explained by the fact that physical activity is an effective intervention in reducing adiposity [20]. The inverse relationship between physical activity and EAT is consistent with the literature reporting a decrease in visceral adipose tissue in response to physical activity [21, 22]. It is similar to some studies in the literature showing reductions in ectopic liver fat and visceral adiposity with even very low levels of physical activity [20, 23]. In the World Health Organization’s physical activity and sedentary time 2020 guideline, the recommendation for physical activity in all age groups to reduce adiposity was expressed as critically important [24]. There are no studies in the literature investigating the relationship between physical activity level and EAT thickness. However, different exercise interventions ranging from moderate to high intensity in various patient populations with increased CVD risk were found to be effective in reducing EAT and improving metabolic parameters [1, 13, 25]. Another important finding of our study, namely the negative correlation between the physical performance levels of hypertensive individuals and EAT thickness, may have been due to the complex interaction of factors such as increased levels of inflammatory mediators and insulin resistance as a result of increased EAT thickness and decreased muscle function [25]. In addition, increased EAT thickness has been associated with lower exercise capacity [26].

There are no studies in the literature investigating the relationship between daily sitting time and EAT thickness. In our study, no relationship was found between EAT thickness and sitting time, in contrast to the positive relationship found between sitting time and pericardial fat by Larsen et al. [27]. This difference may be because our study was conducted in a smaller sample with a different ethnic origin. In addition, in a study analyzing the relationship between sedentary time and visceral adiposity, no relationship was found between these two parameters [22].

Another finding of our study was a positive correlation between EAT thickness and age and waist circumference. No significant correlation was found between EAT thickness and BMI or hip circumference. In the study conducted by Gorter et al. in heart failure patients, higher BMI was directly associated with increased EAT [26]. In the study by Pierdomenico et al. there was no significant relationship between EAT and age, BMI, or waist circumference [28]. Differences between the associations may have been due to population differences in the studies. In some studies, reduction in BMI with diet-based interventions was significantly associated with decreased EAT [29].

Finally, there are still many questions to be answered about sedentary behavior, physical activity, physical performance, and EAT. An important limitation of the available data is that physical activity and sedentary time are measured by self-report through scales and not further validated. Future research is recommended to determine whether these relationships are also observed when using objective measures of activity and sitting. The fact that this is the first study to examine the relationship between sedentary behavior, physical activity, physical performance, and EAT in hypertensive individuals limits interpretation of the findings, and further studies with a larger sample group are needed to confirm these relationships.

## Conclusions

In the present study EAT thickness was negatively associated with physical activity and physical performance in hypertensive patients, but not with daily sitting time. This result shows the importance of the effect of being physically active on blood pressure and EAT. Based on these results, lifestyle change can be emphasized, and rehabilitation can be supported in chronic disease management and prevention.

## Summary

### What is known about topic:

- Epicardial adipose tissue (EAT), which is considered a risk factor for coronary artery disease, is also associated with high blood pressure.
- Recent clinical and experimental studies contain evidence that physical activity (PA) is effective in reducing EAT.

### What this study adds:

- EAT decreases as PA level and physical performance increase in individuals with hypertension. This outcome highlights the significance of the connection between PA and EAT.
- Therefore, it may be beneficial for healthcare professionals to encourage hypertensive patients to adopt a more active lifestyle and to provide support for rehabilitation as part of a comprehensive approach to managing and preventing chronic diseases.

## Data Availability

Data sharing is not applicable to this article as no new data were created or analyzed in this study.
